# Why do physicians prescribe dialysis? A prospective questionnaire study

**DOI:** 10.1371/journal.pone.0188309

**Published:** 2017-12-20

**Authors:** James Heaf, Aivars Petersons, Baiba Vernere, Maija Heiro, Johan V. Povlsen, Anette Bagger Sørensen, Mai Rosenberg, Niels Løkkegaard, Fabiola Alonso-Garcia, Jan Dominik Kampmann, Naomi Clyne, Else Randers, Olof Heimburger, Bengt Lindholm

**Affiliations:** 1 Department of Medicine, Zealand University Hospital, Roskilde, Denmark; 2 P. Stradins University Hospital, Riga, Latvia; 3 University Hospital of Turku, Turku, Finland; 4 Department of Nephrology, Aarhus University Hospital, Aarhus, Denmark; 5 University Hospital of Tartu, Tartu, Estonia; 6 Department of Medicine, Holbaek Hospital, Holbaek, Denmark; 7 Department of Nephrology, Hospital of Southern Jutland, Soenderborg, Denmark; 8 Department of Nephrology in Lund, Clinical Sciences Lund, Skåne University Hospital and Lund University, Lund, Sweden; 9 Department of Medicine, Viborg Regional Hospital, Viborg, Denmark; 10 Department of Clinical Science, Intervention and Technology, Karolinska Institutet, Stockholm, Sweden; Istituto Di Ricerche Farmacologiche Mario Negri, ITALY

## Abstract

**Introduction:**

The incidence of unplanned dialysis initiation (DI) with consequent increased comorbidity, mortality and reduced modality choice remains high, but the optimal timing of dialysis initiation (DI) remains controversial, and there is a lack of studies of specific reasons for DI. We investigated why and when physicians prescribe dialysis and hypothesized that physician motivation for DI is an independent factor which may have clinical consequences.

**Methods:**

In the *Peridialysis study*, an ongoing multicenter prospective study assessing the causes and timing of DI and consequences of unplanned dialysis, physicians in 11 hospitals were asked to describe their primary, secondary and further reasons for prescribing DI. The stated reasons for DI were analyzed in relation to clinical and biochemical data at DI, and characteristics of physicians.

**Results:**

In 446 patients (median age 67 years; 38% females; diabetes 25.6%), DI was prescribed by 84 doctors who stated 23 different primary reasons for DI. The primary indication was clinical in 63% and biochemical in 37%; 23% started for life-threatening conditions. Reduced renal function accounted for only 19% of primary reasons for DI but was a primary or contributing reason in 69%. The eGFR at DI was 7.2 ±3.4 ml/min/1.73 m^2^, but varied according to comorbidity and cause of DI. Patients with cachexia, anorexia and pulmonary stasis (34% with heart failure) had the highest eGFR (8.2–9.8 ml/min/1.73 m^2^), and those with edema, “low GFR”, and acidosis, the lowest (4.6–6.1 ml/min/1.73 m^2^). Patients with multiple comorbidity including diabetes started at a high eGFR (8.7 ml/min/1.73 m^2^). Physician experience played a role in dialysis prescription. Non-specialists were more likely to prescribe dialysis for life-threatening conditions, while older and more experienced physicians were more likely to start dialysis for clinical reasons, and at a lower eGFR. Female doctors started dialysis at a higher eGFR than males (8.0 vs. 7.1 ml/min/1.73 m^2^).

**Conclusions:**

DI was prescribed mainly based on clinical reasons in accordance with current recommendations while low renal function accounted for only 19% of primary reasons for DI. There are considerable differences in physicians´ stated motivations for DI, related to their age, clinical experience and interpretation of biochemical variables. These differences may be an independent factor in the clinical treatment of patients, with consequences for the risk of unplanned DI.

## Introduction

In patients with chronic kidney disease (CKD), the mortality risk increases when glomerular filtration rate (GFR) falls and becomes very high when GFR is below 15 ml/min/1.73m^2^. But, it is not clear to what extent the high mortality risk in CKD stage 5 (CKD5) can be reduced by earlier dialysis initiation (DI), and the precise cause(s) which in clinical practice motivates chronic DI in patients with CKD5 at a particular time are not well studied. Thus, the optimal timing and motivation of DI remains controversial. In the late 1990s, in the hope of preventing malnutrition and avoiding increased mortality and complications linked to too late start of dialysis therapy, estimated GFR (eGFR) at DI increased markedly [1–3]. According to the United States Renal Data System (USRDS), the proportion of patients with eGFR>10 ml/min at DI increased from 20% in 1996 to 52% in 2008. However, the benefits of “early dialysis start” was challenged as the results of several large observational studies comparing outcomes in patients starting dialysis at various levels of eGFR showed that starting dialysis at lower levels of eGFR (= “late start”) associated with lower mortality. In 2010, the IDEAL study [4] showed that in 828 patients randomized to early (eGFR 10–14 ml/min) or late (eGFR 5–7 ml/min) dialysis start, there was no difference in survival or complications between the two groups. On the other hand, 76% of the patients allotted to late dialysis had to start dialysis before the target of <7 ml/min due to uremic symptoms. This led to recommendations that dialysis primarily be initiated when patients become symptomatic [5], or if the patient has severe renal insufficiency, <5 ml/min (reference 5) or <6 ml/min [6]. Thus, the concept of terminal uremia, defined as advanced renal failure requiring permanent active treatment in order to prevent death, disease or invalidity, is now limited to the continued presence of uremic symptoms, life-threatening uncontrollable electrolyte disturbances, and/or severe renal failure. These recommendations have stopped the accelerating trend towards earlier and earlier initiation of dialysis based on eGFR.

A further problem with the IDEAL study is that patients were referred early to specialist nephrological care, were closely monitored, and, as participants in a randomized controlled trial, may have been atypical of the general terminal uraemia population. Probably as a result of these factors, the incidence of urgent DI, defined as need for a temporary dialysis catheter placement, was very low (3.7% for the early start and 8.3% for the late start group respectively) compared with the incidence of about 40% in most other clinical studies [7]. Urgent/unplanned DI is undesirable since it is associated with a substantially increased risk of septicemia [8,9] and mortality [10,11]. While one cause for urgent/unplanned DI is delayed referral of patients with CKD stage 4–5 to specialist nephrological care [12,13], the physicians´ motivation for starting dialysis has received little scientific attention. An international questionnaire survey in 2000 [14], showed that 60% of physicians believed that clinical problems were the most important factors for starting dialysis (nutritional status 14%, overhydration 8%, uremic symptoms 38%), while 32% believed residual renal function (RRF) to be most important. Only 4% did not believe that early start had any clinical benefit. These figures would be expected to be radically different in the post-IDEAL era. However, an international questionnaire study in 2012 [15] showed that a third of nephrologists considered RRF to be most important, rising to 54% for uncomplicated patients. The median eGFR requiring dialysis for uncomplicated patients was 10 ml/min, varying between 5–20 ml/min, suggesting that many physicians remain unconvinced of the implications of the IDEAL study. While the European Renal Best Practice (ERBP) guidelines recommend the mean of urea and creatinine clearance as the optimum method of evaluating RRF [5], most physicians—likely for practical reasons—continue to prefer eGFR, albeit often in combination with another method. There is a continuing uncertainty about the optimum timing of DI, the role of GFR and on what other grounds physicians (should) initiate dialysis.

The Peridialysis project is an ongoing, multi-center, multinational prospective epidemiological study investigating clinical practices up to DI and their immediate consequences (the “peridialysis” period). In the present study, which is the first report from the project, we report data concerning specific causes and timing of DI, focusing on physicians´ stated motivations for prescribing dialysis, and correlations of causes and timing of DI with physician details. We hypothesized that physician motivation for DI is a psychological/sociological phenomenon, independent of other clinical and biochemical variables that may influence DI practices. In particular, we hypothesized that some physicians will prescribe dialysis on mainly clinical grounds, while others will prescribe on mainly biochemical grounds. This is an important difference, since it is possible that differences in physician motivation will affect the patient’s prognosis and choice of dialysis modality.

## Materials and methods

Eleven nephrology departments took part in this observational prospective questionnaire study of causes and timing of DI. All delivered both peritoneal dialysis and hemodialysis. All centers were publicly financed, with no dialysis costs to the patient, but with varying financial support for medicine costs. The commonest method of assessing RRF and guiding clinical treatment was eGFR as measured by the CKD-EPI formula.

### Patients

All consecutive patients starting chronic dialysis therapy for ESRD between 1.1.2015 and 1.1.2016 at the participating centers were included in the current study. Some centers provided additional data up to 1.7.2016. The patient was considered as having end-stage renal disease (ESRD) at first dialysis if

1. The treating physician “believed” that the patient had ESRD at first dialysis
2. The patient received >90 days dialysis treatment
3. If the doctor was in doubt whether the patient had acute or chronic renal failure, the patient was included retrospectively as soon as there was no doubt that the patient had ESRD

The current study comprises 446 patients (median age of 67 years; 38% females) with CKD5 who were investigated in conjunction with DI. Patient data are shown in Table 1. The underlying renal diagnoses were: glomerulonephritis 19.3%, chronic interstitial nephropathy/obstructive 9.4%, polycystic disease 7.4%, type 1 diabetes mellitus (DM) 6.3%, type 2 DM 17.3%, hypertensive nephropathy 17.9%, other 10.8% and unknown 11.7%.

**Table 1:** Clinical and laboratory characteristics at the time of dialysis initiation in 446 patients with CKD5.

	Mean ±SD	Median (IQ range)
Age (years)	64.2 ±14,7	67.0 (55–75)
Females (n; %)	170; 38.0	
Height (cm)	172 ±10	172 (165–179)
Body weight (kg)	79.4 ±21.7	75 (65–90)
Body weight non-diabetics	76.7 ±18.6	74 (64–87)
Body weight diabetics	88.6 ±28.2	83.5 (70–101)
Body mass index (kg/m^2^)	26.5 ±6.1	25.0 (22.5–29.3)
BMI non-diabetics	26.0 ±5.6	24.7 (22.3–28.4)
BMI diabetics	28.3 ±7.1	27.5 (23.2–30.9)
Number of comorbidities	1.39 ±1.4	1 (0–2)
*Biochemical measurements*		
Creatinine (μM)	689 ±303	631 (508–797)
Hemoglobin (mM)	6.3 ±1.1	6.2 (5.6–6.9)
Potassium (mM)	4.5 ±0.8	4.4 (3.9–4.9)
Urea (mM)	33.7 ±11.9	31.8 (25.6–39.7)
Bicarbonate (mM)	20.9 ±5.2	21.0 (17.6–24.8)
Albumin (mM)	33.8 ±6.7	34 (29–39)
C-reactive protein (mg/l)	38 ±59	10 (3–49)
Ionized calcium (mM)	1.14 ±0.12	1.14 (1.07–1.21)
Phosphate (mM)	1.99 ±0.56	1.95 (1.60–2.31)
eGFR (ml/min/1.73 m^2^)	7.2 ±3.4	6.7 (5.0–8.7)
eGFR 3 months previously	14.0 ±13.6	10.1 (8.5–13.5)
Change in eGFR (ml/min/1.73 m^2^/yr)	*	10.3 (3.7–21.6)
*Comorbidities*		
	**Percent**	
Myocardial infarction	11.2	
Cardiac failure	16.1	
Cardiac disease	23.8	
Cerebrovascular disease	12.6	
Peripheral vascular disease	13.0	
Diabetes	25.6	
Cancer	13.7	
Pulmonary disease	11.2	
Hepatic disease	4.3	
Psychiatric disease	4.7	
Previous transplant	2.9	
No comorbidity	19.7	

*Not stated; a minority of patients had extreme values.

The study protocol was approved by the ethical review boards in centers located in countries where according to the country´s regulations such perusal was required. The study was approved by the Swedish Ethical Committee (Ref 2017/7). However, in Denmark, due to the observational non-interventional design of the study using anonymized patient data, the study protocol was not considered to be eligible for ethical review. Informed consent—either written or verbal depending on the regulations in the different countries—was obtained from participants in all centers including those in Denmark The study is registered with Clinical Trials.gov, identifier NCT02488200

The datafile for this project is available on Open Science Framework, identifiers: DOI 10.17605/OSF.IO/Z63JP, ARK c7605/osf.io/z63jp

### Methods

#### Patient clinical data

The following patient data at DI were registered: age, sex, height, weight, body mass index (BMI) and renal diagnosis. The presence of the following comorbidities was registered: previous myocardial infarction, heart failure, cardiac atherosclerosis, cerebrovascular disease, diabetes, peripheral atherosclerosis, previous cancer (except basocellular), chronic pulmonary disease, chronic liver disease, psychiatric disease, previous renal transplantation, and other specified chronic conditions.

#### Patient biochemical data

The following biochemical data prior to or in conjunction with first dialysis were registered: blood hemoglobin, plasma values of urea, creatinine, potassium, hydrogen carbonate (bicarbonate), albumin, C-reactive protein (CRP), total or ionized calcium, and phosphate. Most centers measured ionized calcium, for other centers, ionized calcium was assumed to be 50% of total calcium.

#### Physician motivation questionnaire

Physicians gave details of their reasons for prescribing chronic dialysis in an English language questionnaire (S1 Table). They could choose between clinical and/or biochemical reasons. Since the terms used in the questionnaire were deemed to be clearly defined, no questionnaire validation for local translations were performed.

*Clinical reasons*: pulmonary stasis, dyspnea, hypertension, pericarditis, oedema, cardiac symptoms, fatigue, anorexia, nausea/vomiting, cachexia/weight loss, itching, insomnia, depression, diarrhea, taste disturbances, social problems, practical problems, and other specified reasons.

*Biochemical reasons (based on plasma values)*: high creatinine, high urea, low GFR, high potassium, acidosis, low calcium, high calcium, high phosphate, falling GFR.

A primary reason for DI had to be specified, and a secondary was voluntary. In addition, other reasons for DI could be stated by the physician who also was encouraged to register the presence of uremic symptoms at dialysis prescription.

#### Physician data

Data on participating physicians (n = 84) were requested and for those who agreed to have their data registered (n = 52, contributing to 74% of DIs)) data were anonymized. They were asked to provide the following personal data: age, sex, specialist qualification, duration of physician experience, and duration of specialist nephrology experience.

## Statistics

Data are presented as mean ± standard deviation (SD) or median (interquartile, IQ, range) or as numbers (percentage). Parametric variables were compared using the Students t-test and MANOVA, and non-parametric using the Chi square and Kruskal-Wallis tests. A probability level of <0.05 was considered significant. Significance values were expressed as p<0.05, p<0.01, p<0.001. For comparisons, variables were divided according to median value or as tertiles.

## Results

**Fig 1:**
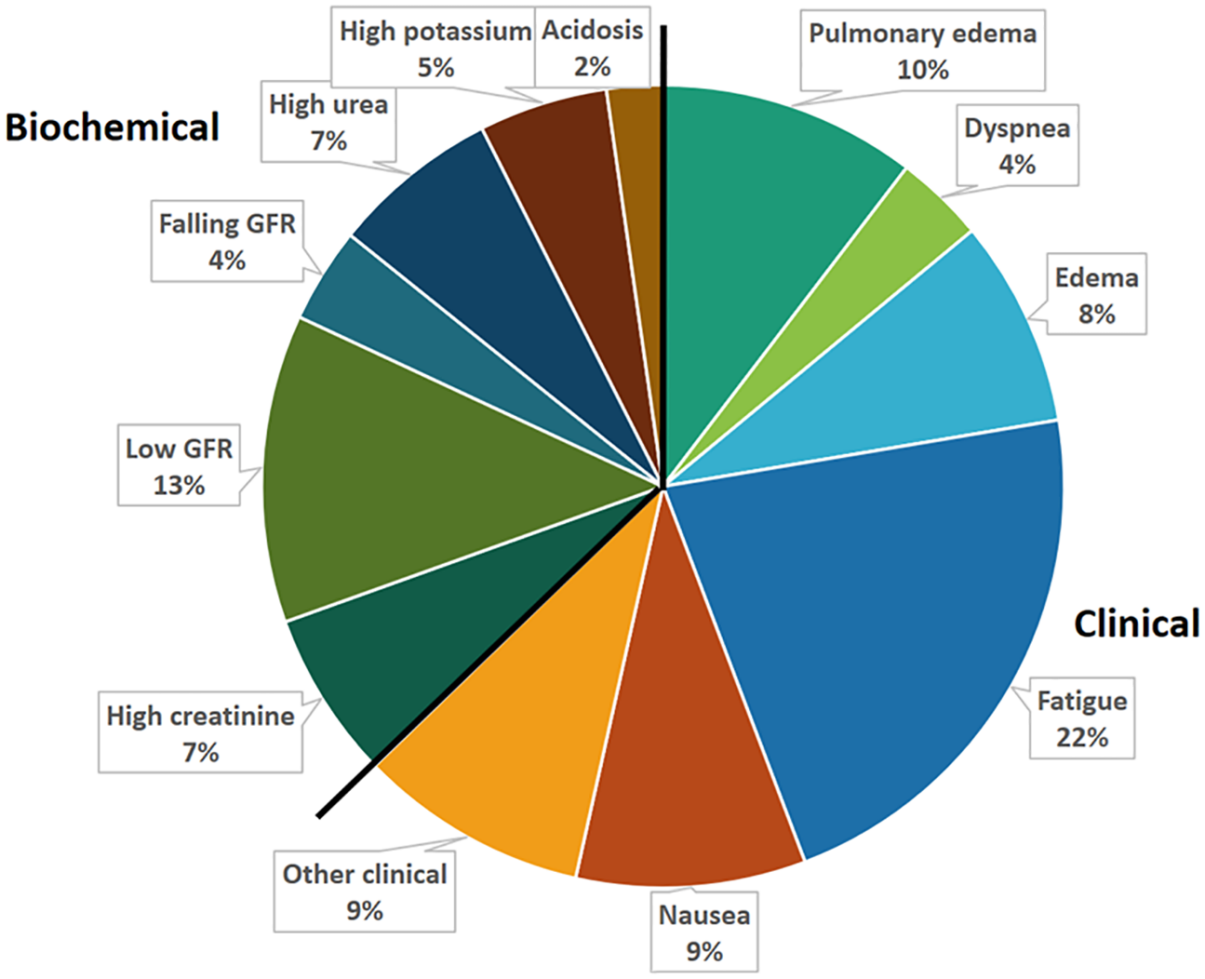
Primary reasons for chronic dialysis initiation (DI) in 446 patients with CKD5. Clinical reasons accounted for 63% and biochemical reasons for 37% of all DI. The 12 most common motivations accounted for 91% of all DI prescriptions, “fatigue” being most common (22%) among clinical motivations, and “low GFR” (13%) most common among DI based on biochemical grounds.

**Fig 2:**
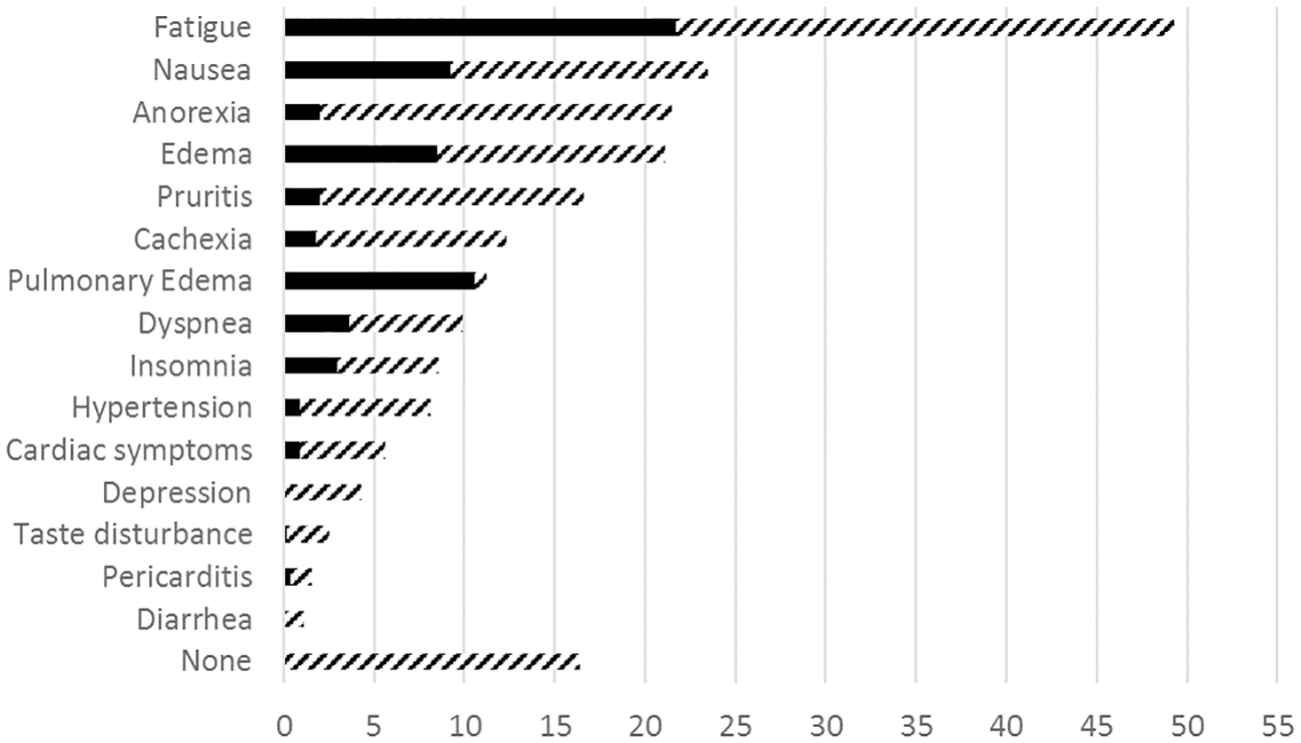
Uremic symptoms at dialysis initiation (DI) in 446 patients with CKD5 in percent. Symptoms were present in 83% of patients, the most common being fatigue (44%), nausea (24%) and anorexia (22%). Black bars: primary symptoms; hatched bars: secondary or other symptoms.

Dialysis initiation (DI) in 446 patients was prescribed by 84 doctors at 11 hospitals. Patient data are shown in Table 1. The reasons (19 clinical and 11 biochemical) for prescribing dialysis are shown in Table 2 and Fig 1 and clinical symptoms in Fig 2. The primary reason for DI was clinical in 62.7% of cases, 16.4% had no biochemical indication, and 16.9% no clinical indication. 48.0% had 1–2 symptoms, 21.5% 3–4 and 13.6% >4 symptoms. Life threating conditions (pulmonary stasis, dyspnea, pericarditis, cardiac symptoms, tetanus, hyperkalemia and acidosis) were the primary reason in 102 (22.9%) of cases. All 19 pre-stated clinical symptoms that a priori were assumed to be main causes for DI were present in one or more of patients and “other” clinical causes including ascites, freezing symptoms, muscle cramps, tetanus and vertigo were also noted in some of the patients. Low renal function (high creatinine or low eGFR) was the primary cause in 19.1% but low RRF was noted as a factor prompting DI prescription in altogether 69.4% of the cases. Only 14 patients (3.1%) had an eGFR >15 ml/min at DI.

**Table 2:** Primary, secondary and additional other reasons, clinical and biochemical, for prescribing chronic dialysis initiation in 446 patients.

	Primary		Secondary		Other		Total	
	No.	Percent	No.	Percent	No.	Percent	No.	Percent
**Clinical**								
Pulmonary edema	46	10.3	2	0.2	2	0.4	50	11.2
Dyspnea	16	3.6	4	0.9	24	5.4	44	9.9
Hypertension	4	0.9	5	1.1	27	6.1	36	8.1
Pericarditis	2	0.4	1	0.2	4	0.9	7	1.6
Edema	37	8.3	16	3.6	40	9.0	92	21.1
Cardiac symptoms	4	0.9	8	1.8	13	2.9	25	5.6
Fatigue	97	21,7	43	9.6	80	17.9	220	49.3
Anorexia	9	2.0	25	5.6	62	13.9	96	21.5
Nausea	41	9.2	23	5.2	41	9.2	105	23.5
Cachexia/weight loss	9	1.8	10	2.2	36	8.1	55	12.3
Pruritis	9	2.0	13	2.9	52	11.7	74	16.6
Insomnia	0	0	3	0.7	22	4.9	25	5.6
Depression	0	0	2	0.4	17	3.8	19	4.3
Diarrhea	0	0	0	0	5	1.1	5	1.1
Taste disturbances	1	0.2	0	0	10	2.2	11	2.5
Cerebral symptoms	1	0.2	0	0	4	0.9	5	1.1
Social	0	0	0	0	5	1.1	5	1.1
Practical	3	0.7	1	0.2	1	0.2	5	1.1
Other	1	0.2	0	0	9	2.0	10	2.2
*Any clinical*	280	62.7	156	35.0	234	52.5	373	83.6
*Life-threatening*	102	22.9					178	40.0
**Biochemical**								
High creatinine	30	6.7	26	5.8	101	22.6	157	35.2
Low eGFR	55	12.3	32	7.2	99	22.2	186	41.7
High creatinine or low eGFR	85	19.1					265	69.4
Falling eGFR	17	3.8	34	7.6	62	13.9	113	25.3
High urea	30	6.7	37	8.3	73	16.4	140	31.4
High potassium	23	5.2	13	2.9	25	5.6	60	13.5
Acidosis	10	2.2	17	3.8	47	10.5	74	16.6
Low calcium	0	0	2	0.4	13	2.9	15	3.4
High calcium	0	0	0	0	2	0.4	2	0.4
High phosphate	1	0.2	0	0	49	11.0	50	11.2
*Any biochemical*	166	37.2	161	36.1	228	51.1	371	83.1

Among the 84 prescribing physicians, 52 physicians, responsible for 332 (74.4%) of prescriptions, supplied their personal details. Their age was 49 ±10 years, median 49 (interquartile, IQ, range 41–58) years; and 27 (54%) were female. They had been qualified physicians for 22 +/- 10 years, median 22 (13–45) years, and 41 (79%) were specialist nephrologists, and had been so for on average 13 ±9 years, median 13 (6–19) years. Females physicians were generally younger (45.8 ±9.1 vs. 52.0 ±9.8 years, p<0.05). Fewer females were specialists (67% vs. 89%), and fewer had long specialist experience (>13 years, 21% vs. 54%) as compared to the males.

There were considerable differences in the behavior of physicians, as shown in Table 3. Older physicians were more likely to prescribe dialysis on primarily clinical grounds, but were also more likely to include biochemical reasons in their prescription. Females were more likely to initiate dialysis for primarily life-threatening reasons. Non-specialists were more likely to include clinical grounds in their reasoning, and to start dialysis for life-threatening reasons. Physicians with more specialist experience were more likely to give clinical reasons, and less likely to give life-threatening reasons. There were no significant differences between the prescribers in eGFR at first dialysis, though there was an insignificant trend for more experienced doctors to start at a lower renal function, and total physician experience showed a similar pattern to specialist experience.

**Table 3:** The stated primary reason for dialysis initiation in 446 patients and characteristics of prescribing physicians (n = 52) who agreed to provide their details. Number and percent (in brackets).

	eGFR	Primary reason		Clinical reasons		Biochemical reasons		1° Life-threatening reason	
	ml/min/1.73 m^2^	Clinical	Biochemical	None	Some	None	Some	Yes	No
**Age (yrs)**									
<50	7.8 ±.9	71 (51.4)***	67	30 (21.7)**	108	13 (8.8)***	125	38 (27.5)*	100
>49	7.3 ±3.3	139 (71.6)	55	21 (10.8)	173	47 (24.2)	147	35 (18.4)	154
**Sex**									
Male	7.2 ±3.5	134 (62.6)	80	32 (14.9)	182	43 (20.1)	171	35 (16.4)***	179
Female	8.0 ±3.6	76 (64.4)	42	19 (16.1)	99	17 (14.4)	101	38 (32.2)	80
**Medical experience**									
<23 yrs	7.9 ±3.9	71 (51.8)***	66	30 (21.9)**	107	13 (9.5)***	124	38 (27.7)*	99
>22 yrs	7.2 ±3.3	139 (71.3)	56	21 (10.7)	174	47 (24.1)	148	35 (18.0)	160
**Specialist experience**									
None^a^	8.0 ±4.8	31 (66.0)	16	2 (4.2)*	45	6 (12.7)	41	18 (38.3)**	29
<14 yrs^b^	7.6 ±3.5	64 (51.2)***	61	34 (27.2)***	91	16 (12.8)*	109	32 (25.6)*	93
>13 yrs	7.3 ±3.1	115 (71.8)	45	15 (9.3)	145	38 (23.8)	122	23 (14.4)	137

*:p<0.05;

**:p<0.01;

***:p<0.001;

^a^: significance values compared to specialists;

^b^:compared to specialists with more experience

When excluding patients with primary life-threatening reasons to take into account the possibility that less experienced doctors were more likely to meet acutely ill patients, the results were generally unchanged. However, female physicians started dialysis at a higher eGFR than male doctors (8.0 ±3.6 ml/min vs. 7.2 ±3.5, p<0.05).

The biochemical relationship to the biochemical reasons given for starting dialysis are shown in Table 4. There was general agreement on the interpretation of the terms, with a considerable overlap. Patients with a “high creatinine” had a slightly higher creatinine than those without, but there was no significant difference in the corresponding eGFR. Similarly, patients with a “low GFR”, had a significantly lower eGFR, but no difference in creatinine. The choice of the terms “high creatinine” or “low eGFR” as primary reason did not relate to physician details. There was no agreement on the meaning of the term eGFR.

**Table 4:** Biochemical primary reasons for dialysis initiation in 446 patients as stated by prescribing physicians (n = 84) and how these reasons relate to the corresponding measurements in presence or absence of stating these reasons as a cause to start dialysis.

Term	Present			Absent			P
	No.	Mean ±SD	Median (IQ range)	No.	Mean ±SD	Median (IQ range)	
High creatinine (μM)	30	781 ±333	707 (545–947)	282	675 ±343	635 (495–790)	(0.06)
(corresponding eGFR (ml/min/1.73 m^2^))		6.5 ±3.3	5.6 (4.3–8.4)		7.2 ±3.5	6.6 (5.0–8.7)	NS
Low eGFR (ml/min/1.73 m^2^)	51	6.1 ±2.1	5.9 (4.8–7.4)	251	7.6 ±3,7	6.9 (5.1–9.4)	<0.01
(corresponding creatinine (μM))		719 ±258	669 (576–825)		678 ±317	627 (575–825)	NS
Falling eGFR (ml/min/1.73 m^2^/yr	11		9.2 (2.4–47.6)	238		11.7 (4.3–22.5)	NS
High urea (mM)	30	42.2 ±15.6	39.6 (33.0–49.2	298	32.0 ±11.2	29.5 (24.9–38.5)	<0.001
High potassium (mM)	22	5.7 ±1.3	6.0 (4.5–6.5)	364	4.3 ±0.7	4.3 (3.9–4.8)	<0.001
Acidosis (mM)	10	16.0 ±8.7	13.6 (10–19)	305	21.7 ±4.9	22 (18–25)	<0.001
Low calcium*	15	0.96 ±0.11	0.96 (0.86–1.02)	402	1.14 ±0.12	1.15 (1.08–1.22)	<0.001
High phosphate*	44	2.17 ±0.57	2.04 (1.83–2.33)	367	1.97 ±0.55	1.92 (1.56–2.30)	<0.05

*primary, secondary and other reasons combined.

We hypothesized that the following variables would correlate to clinical uraemia: eGFR, BMI, albumin, bicarbonate, and CRP. The data are shown in Table 5. There were considerable differences in the eGFR for the various primary problems. Patients with cachexia, anorexia and pulmonary stasis had an eGFR of 8.2–9.8 ml/min, while patients with acidosis and edema were characterized by a low eGFR (4.6–6.1 ml/min). BMI was broadly similar, but cachectic patients had as expected a lower level, on average 21.3 kg/m^2^. Conditions characterized by hypoalbuminemia (≤33 g/l) included pulmonary stasis, hyperkalemia, dyspnea, edema and acidosis. A low bicarbonate (≤20 mM) was seen in patients with hyperkalemia, and in those with a rapidly falling eGFR, or cardiac symptoms. Pulmonary stasis, dyspnea, acidosis and hyperkalemia were generally associated with inflammation (CRP>24 mg/l). Patients with primarily clinical grounds for DI and/or no biochemical reasons had a significantly higher eGFR. Patients with life-threatening conditions were generally biochemically abnormal, with hypoalbuminemia, acidosis and a raised CRP.

**Table 5:** Biochemical correlates to primary reasons, ranked according to eGFR.

	eGFR (ml/min/1.73 m^2^)	BMI (kg/m^2^)^a^	Albumin (g/l)	Bicarbonate (mM)	C-reactive protein (mg/l)^b^
Cachexia	9.8 ±5.7	21,3 ±3.3	34,8 ±3.3	21,9 ±6.4	3 (1–3)
Pericarditis	8.9 ±3.8	28,1 ±1.8	31,0 ±5.7	24,0 ±8.5	158 (-)
Anorexia	8.4 ±1.8	27,7 ±6.2	36,8 ±7.1	22,2 ±3.3	7 (3–17)
Pulmonary stasis	8.2 ±4.8	27,6 ±7.6	32,0 ±6.5	20,5 ±5.7	38 (9–91)
Falling eGFR	8.1 ±3.5	23,2 ±3.7	34,8 ±7.1	19,4 ±3.4	22 (4–72)
Itching	7.9 ±3.4	24,8 ±4.5	34,1 ±9.8	21,6 ±2.6	24 (5–37)
Fatigue	7.9 ±3.5	26,8 ±5.7	35,0 ±6.2	21,6 ±4.9	5 (3–25)
Cardiac symptoms	7.5 ±4.8	25,4 ±4.4	35,7 ±4.9	19,0 ±2.6	20 (1–139)
High potassium	7.3 ±3.3	24,9 ±7.9	32,4 ±7.5	18,6 ±4.9	25 (4–75)
High urea	7.2 ±3.5	24,6 ±4.0	33,5 ±5.4	20,8 ±5.7	14 (3–60)
Nausea	7.0 ±3.3	24,2 ±5.0	32,9 ±8.0	20,8 ±4.6	3 (3–33)
High creatinine	6.5 ±3.3	25,0 ±4.4	35,1 ±6.4	20,3 ±5.8	12 (10–146)
Dyspnea	6.3 ±2.3	29,0 ±6.7	31,2 ±7.1	19,9 ±6.6	65 (10–146)
Hypertension	6.3 ±2.6	31,0 ±9.4	36,3 ±1.5	19,0 ±2.0	9 (2–24)
Edema	6.1 ±2.0	25,9 ±5.2	30,1 ±6.1	20,3 ±5.5	19 (3–86)
Low eGFR	6.1 ±2.1	25,8 ±4.7	36,5 ±6.2	22,4 ±4.1	5 (3–20)
Acidosis	4.6 ±2.3	27,6 ±6.1	31,1 ±7.9	16,0 ±8.7	29 (3–69)
**Motivational groups**					
Primary Clinical	7.5 ±3.6**	26.3 ±5.9	33.4 ±6.7	21.1 ±5.1	10 (3–54)
Primary Biochemical	6.6 ±3.0	25.2 ±5.1	34.7 ±6.6	20.6 ±5.4	11 (3–44)
Life-threatening	7.3 ±4.0	26.2 ±5.9	31.9 ±6.8***	19.5 ±6.1***	37 (5–91)***
Not life-threatening	7.4 ±3.3	25.7 ±5.4	35.1 ±6.6	22.0 ±4.7	6 (3–30)
No clinical	7.1 ±3.1	26.0 ±5.7	34.9 ±7.2	20.6 ±5.4	9 (3–53)
Some clinical	7.2 ±3.5	25.8 ±5.7	33.6 ±6.6	21.0 ±5.1	11 (3–49)
No biochemical	8.2 ±3.6**	25.0 ±5.6	34.2 ±6.7	20.5 ±5.2	12 (3–86)
Some biochemical	7.0 ±3.3	26.0 ±5.7	33.7 ±6.7	21.0 ±5.2	9 (3–45)
All	7.2 ±3.4	25.8 ±5.7	33.8 ±6.7	20.9 ±5.2	10 (3–49)

^a^excluding patients with type 2 DM;

^b^: Median (IQ range);

**:p<0.01;

***:p<0.001

Correlations to renal diagnosis, patient sex and age are shown in Table 6. eGFR was not related to diagnosis, but in a post hoc analysis, eGFR was higher in patients with diabetic nephropathy (p<0.05). Diabetics were more likely to start dialysis on clinical indications. Patient sex was not related to eGFR or physician motivation. Younger patients (<60 years) were more likely to start because of life-threatening conditions.

**Table 6:** Correlations to renal diagnosis, patient sex and patient age. No. patients (%).

	eGFR	Primary reason		Clinical reasons		Biochemical reasons		1° Life-threatening reason	
	ml/min/1.73 m^2^	Clinical	Biochemical	None	Some	None	Some	Yes	No
**Diagnosis**									
Diabetic	7.8 ±3.2*^a^	81(77.1)***^b^	24	11 (10.5	94	19 (18.1)	86	27 (25.7)	78
Polycystic	7.6 ±2.5	17 (51.5)	16	4 (12.1)	29	7 (21.2)	26	8 (24.2)	25
Unknown	7.1 ±2.7	31 (59.6)	21	9 (17.3)	43	9 (17.3)	43	12 (23.1)	40
Chronic interstitial	7.1 ±3.5	26 (61.9)	16	9 (21.3)	33	7 (16.7)	35	10 (23.8)	32
Other	7.1 ±5.7	27 (56.3)	21	9 (18.8)	39	5 (10.4)	43	15 (31.2)	33
Hypertesnive	7.0 ±2.8	48 (60.0)	32	16 (20.0)	64	15 (18.8)	65	15 (18.7)	65
Glomerulonephritis	6.7 ±3.2	50 (58.1)	36	14 (16.3)	72	13 (15.1)	73	15 (17.4)	71
**Sex**									
Male	7.4 ±3.5	171 (62.0)	105	42 (15.2)	234	49 (17.8)	227	70 (25.3)	206
Female	7.0 ±3.2	109 (64.5)	60	30 (17.8)	139	26 (15,4)	143	32 (18.9)	137
**Age (years)**									
<60	7.1 ±3.8	94 (63.1)	55	21 (14.1)	128	17 (11.4)	132	26 (17.5)*^c^	123
60–71	7.4 ±3.3	91 (64.5)	50	23 (16.3)	118	31 (22.0)	110	34 (24.1)	107
>71	7.1 ±3.2	91 (61.1)	58	26 (17.5)	123	27 (18.1)	122	42 (28.1)	107

*:p<0.05;

***:p<0.001;

^a^: see text;

^b^: versus other diagnoses;

^c^: versus other age groups.

The presence of comorbidity had a major influence on biochemistry and DI (Table 7). Patients with more than three comorbidities started dialysis at an eGFR that was 2.3 ml/min higher than patients with none, 8.7 ±4.7 vs 6.4 ±2.6 ml/min. CRP rose continuously, and albumin fell with increasing comorbidity. A post hoc Spearman correlation analysis revealed albumin and CRP to be highly inversely correlated (R = -0.40, p<0.001). Patients with pulmonary stasis had a significantly higher prevalence of heart failure (34% vs. 14%, p<0.001).

**Table 7:** Proportion of clinical causes as reason for dialysis initiation and selected measurements in relation to number of comorbidities.

No. comorbidities	No.	Primary clinical cause (%)**	eGFR (ml/min/1.73 m^2^)***	BMI (kg/m^2^)^a^	Albumin (g/l)**	Bicarbonate (mM)	C-reactive protein (mg/l)***
0	137	58	6,4 ±2.6	26,0 ±5.5	35,6 ±6.8	20,5 ±5.0	4 (3–26)
1	148	59	7,2 ±3.7	25,6 ±5.8	33,6 ±6.6	21,3 ±5.3	8 (3–35)
2	71	65	7,0 ±2.4	26,6 ±6.2	33,3 ±7.0	21,2 ±5.2	20 (3–71)
3	47	72	8,1 ±3.8	25,3 ±5.8	33,2 ±5.7	19,5 ±5.3	18 (9–76)
>3	43	79	8,7 ±4.7	25,3 ±4.3	31,0 ±6.2	21,4 ±5.0	37 (9–73)

^a^excluding patients with type 2 DM;

**:p<0.01;

***:p<0.001

## Discussion

There are few studies of clinicians’ motivation for prescribing dialysis, and this is the first comprehensive prospective study in the post-IDEAL era. There were 21 primary causes stated by the 84 prescribing physicians as reason for starting dialysis among the 446 patients, illustrating that terminal uraemia is a kaleidoscopic condition. While most patients (63%) started dialysis on primarily clinical grounds, low renal function was the primary reason in only 19% of cases: however, residual renal function was stated as playing a role in 69% of cases. The physicians seem generally to be following the conclusions of the IDEAL study [4] and recommendations from ERBP [5], the Canadian Society of Nephrology [16] and KDOQI [17] that the decision to initiate maintenance dialysis should be based primarily on assessment of signs and/or symptoms of uraemia and related conditions such as fluid overload and secondly in the presence of severe renal failure (GFR <5–6 ml/min). There has been some criticism of the conclusions of the IDEAL study [18]. Clinical uremia was not clearly defined in the study. Fig 2 suggests that the primary uremic symptom is fatigue, and this symptom may not necessarily be alleviated by dialysis [19], particularly if symptoms are caused by comorbid disease or hemodialysis side effects. If the cause of dialysis initiation is non-life-threatening uremic symptoms, the final decision should whenever possible be made by the patient, who must balance symptomatology against the inconvenience of dialysis. There are no financial incentives for early or late start of dialysis in the countries involved in this study; it is possible that the presence of such incentives could lead to suboptimal patient treatment [18].

Previous studies have reported uremic symptoms at DI.[20–23] The methodology of these differed from the present study in a number of ways: two were based upon patient notes,[21,23] one on objective assessment rather than symptoms [20]. In one study patients with acute uremia were excluded [22] and another was limited to nursing home patients[20]. DI in these studies occurred at a higher eGFR, varying from 8.9 ml/min [21] to 11.0 ml/min [23]. O’Hare [23] found a DI eGFR >15 ml/min in 16% of patients, and Kurella 18% [20]. Significant causes of early DI were edema and dyspnea. Since only 3% of our patients started DI at an eGFR >15 ml/min, this suggests that these symptoms can usually be controlled by conservative measures, e.g. diuretics, fluid restriction and sodium bicarbonate supplements.

Seventeen percent of our patients had no uremic symptoms at DI, generally in accordance with other studies: 12% [22] and 18% [20]. However, there is some disagreement concerning the number of symptoms. 22% of our patients had >2 symptoms, compared to 20% in the Kurella study [20] and 61% in the Curtis study [22]. There is general agreement that fatigue, nausea, anorexia, volume overload and itching are the commonest uremic symptoms.

The symptoms of uraemia are often non-specific, and many patients probably started dialysis for reasons other than uraemia, in that patients with multiple comorbidity, including diabetes and with probable symptoms related to these, started dialysis earlier than non-comorbid patients. Similarly, patients with pulmonary stasis started dialysis early. This may have been due to unrelated cardiac failure (in 34% of the cases), but suggests that increased attention to volume control in these patients could have postponed dialysis requirement. Patients starting due to pulmonary stasis and dyspnea had very high values of CRP, suggesting that complicating pneumonia may have been a partial cause of their distress.

One surprising fact is that cachexia and/or weight loss was the primary indication in only 2% of patients. A previous survey [14] showed that nutritional problems were considered to be an important reason for starting dialysis. Plasma albumin has often been used as a marker of malnutrition. This is probably erroneous: in this study, albumin was a marker of increasing morbidity and inflammation as measured by CRP. The interplay between protein malnutrition and inflammation is a complex issue, since albumin is both a marker of nutrition and of inflammation. Patients who are sarcopenic often have increased inflammation (the “malnutrition-inflammation-atherosclerosis syndrome”). This will further have complicated the decision algorithm for physicians prescribing dialysis on mainly biochemical grounds.

Furthermore, patients suffering from pulmonary stasis, dyspnea, or edema were characterized by hypoalbuminemia, suggesting a dilutional effect. Patients who started dialysis on life-threating clinical grounds generally had a lower p-albumin and higher C-reactive protein.

There were considerable differences between doctors according to clinical experience. The higher proportion of prescription of dialysis for life-threatening reasons among non-specialists could be related to their probable closer contact with acutely ill patients. In contrast, older doctors (>49 years) and specialists with >13 years of specialist experience were most likely to state primarily clinical reasons for DI. It is possible that these physicians are those most likely to accept the implications of the IDEAL study. Correspondingly, more experienced physicians tended to start dialysis at a lower eGFR. Differences between male and female doctors were relatively minor, but females started dialysis at a higher eGFR than males, probably due to their higher contact with acutely ill patients.

There appeared to be general agreement on the meaning of the biochemical indications for dialysis, but with considerable overlap. This overlap could either be due to real disagreement about the meaning of the terms, or disagreement about what level indicates DI, but implies that there is a considerable variation among doctors as to the optimal timing of DI. In general, doctors started dialysis purely on the basis of renal function only if the eGFR was about 6 ml/min. All in all, this paper demonstrates considerable confusion among physicians concerning definitions and their reasons to start dialysis.

A priori, one would expect the terms “high creatinine” and “low eGFR” to be equivalent; this was however not the case, in that patients with “high creatinine” at DI had similar eGFR as other patients, and vice versa. The choice of terms was not related to physician experience. They thus seem to be perceived as slightly different concepts, the clinical meaning of this difference being unclear. It may be that “high creatinine” is used by physicians who are skeptical about the meaning of eGFR (vide infra). There was no agreement as to what constituted a rate of loss of eGFR that indicated dialysis start. However, rate of loss of eGFR during the last 1–2 months before dialysis was not registered, and might have revealed significant differences.

There is substantial evidence that eGFR is not a reliable measure of renal function in patients with CKD5. Paradoxically, a high eGFR at DI was reported to associate with increased mortality [1,24–26]. This may be due to the fact that many of these patients are malnourished, with reduced muscle mass and low creatinine production, and as a consequence a “falsely” raised eGFR [24,27,28]. Our results support this hypothesis in that patients with cachexia and anorexia had the highest values of GFR, and support the current recommendations of the ERBP guidelines that the average of creatinine and urea clearances, measured from a 24-hour urine collection, should be the preferred method of assessing renal function in CKD5 [5]. However, in the present study, there was no relationship between eGFR and patient age suggesting that age related alterations in body composition such as sarcopenia did not play a role as modifier of eGFR. Another possible explanation for the paradoxical relationship of higher eGFR with increased mortality is that patients with a high eGFR may have more comorbidity, and therefore start dialysis earlier, as previously described [29]. It is possible that dialysis may not be strictly necessary for these patients, but it is in practice often impossible for the physician to distinguish symptoms of uraemia from other diseases, and, in addition, as mentioned above, comorbid patients are usually malnourished and may have a “falsely” raised eGFR.

There are several limitations, which should be taken into account when interpreting the results of the present study. One important problem that is not addressed by the study is the converse question “Why do physicians not prescribe dialysis?” This question does not involve the question as to whether to withhold active treatment either due to severe comorbidity or due to patient choice, but the fact that all contacts to patients with CKD stage 5 must address this question. The answer might be somewhat different from just the absence of reasons for starting dialysis. The study does not reliably supply insights into the true "why" of the psychology of dialysis start today and not tomorrow. For that aim, qualitative research, or vignette studies, should be better suited. In the current setting, respondents can only provide answers already selected for them. The study was limited to the “peridialysis” period and did not include analysis of patient referral to nephrology care, or GFR trajectories and appearance of symptoms and signs and how they were treated during the months or years preceding DI; such information was most likely considered and should have influenced the decisions by the prescribing physicians. So far, the prospective data on the consequences of timing and motivation of DI are not yet available, and therefore no conclusions can be made as regards the implications of the observed findings. The relatively low number of patients, and the low number of prescribing physicians, reduces the statistical power while the generalizability of the study is confined mainly to the Nordic area where the study was performed. On the other hand, a major strength of the study is that it used a more detailed questionnaire about the causes for DI and that it collected information about the prescribing physicians. Furthermore, the study addresses an area where few recent studies have been conducted.

## Conclusions

In conclusion, this first report from the prospective questionnaire Peridialysis study on timing and causes prompting physicians to prescribe maintenance dialysis shows that dialysis initiation was mainly motivated on clinical grounds whereas eGFR and other indices of renal function loss appear to have had more of a supportive role for their decisions. There were considerable differences in motivations stated by physicians for prescribing dialysis, which are related to their age and clinical experience. These differences may be an independent factor in the clinical treatment of patients with consequences for the risk of unplanned dialysis initiation.

## Supporting information

S1 Table Motivation questionnaires. (DOCX)
