# Supplementary material for: Why do physicians prescribe dialysis? A prospective questionnaire study
Source: PLoS One. 2017 Dec 20;12(12):e0188309. doi: 10.1371/journal.pone.0188309 (PMC5737975; doi:10.1371/journal.pone.0188309)
Supplement: S1 Table — (DOCX) [file pone.0188309.s001.docx]

**S1 Table.** Motivation questionnaires

| **Peridialysis Project** | | | | **Hospital:** | |
| --- | --- | --- | --- | --- | --- |
| Patient Name and Identity Number (Label) |  | | | | Project Code (filled in later by project coordinator) |
| Physician (Initials) |  | | Date of dialysis prescription | |  |
| **Instructions.**  You have just prescribed this patient’s first dialysis.  If you do not belive that the patient’s dialysis requirement is chronic, you do not have to fill in this form  **Put a ”1” besides the primary reason for starting dialysis today.**  Put a ”2” besides a (possible) secondary reason for starting dialysis today.  Put a ”X” besides any other reasons that have been important in influencing your decision to prescribe dialysis today.  **In addition, physicians are encouraged to describe the patient’s various clinical symptoms at dialysis start with “X” where relevant.**  Fill in the questionnaire (with today’s date) even if the first dialysis is prescribed to take place in the future (e.g. “PD training in 3 weeks”)  Feel free to add any comments.  Place your answers in the allotted tray(s) or deliver to XXXXX.  There are no ”right” or ”wrong ” answers. Your answers are confidential.  Fille in the questionnaire, with today’s date, even if the prescribed first dialysis is to take place in e.g. one or three weeks time. | | | | | |
| **Clinical** |  | Taste disturbances | | |  |
| Pulmonary stasis |  | Social (what?) | | |  |
| Dyspnea |  | Practical (what?)  (e.g. catheter protection) | | |  |
| Hypertension |  |  | | |  |
| Pericarditis |  |  | | |  |
| Oedema |  | **Biochemical** | | |  |
| Cardiac symptoms |  | High P-Creatinine | | |  |
| Fatigue |  | High Urea | | |  |
| Anorexia |  | Low GFR | | |  |
| Nausea/vommiting |  | High Potassium | | |  |
| Cachexia/weight loss |  | Acidosis | | |  |
| Itching |  | Low calcium | | |  |
| Insomnia |  | High Calcium | | |  |
| Depression |  | High Phosphate | | |  |
| Diarrhea |  | Falling GFR | | |  |
| Other (what?) | | | | | |

| **Peridialyse Project** | | | | **Hospital** | |
| --- | --- | --- | --- | --- | --- |
| Patient Navn og CPR-nr. |  | | | | Projekt nr. (tilføjes senere af projektansvarlig)  DK |
| Ordinerende Læge (Initialer) |  | | Dato | |  |
| **Instruktioner.**  Du har lige ordineret denne patients første dialyse.  **Sæt et ”1” ved siden af den primære årsag for at ordinere dialyse I dag.**  Sæt et ”2” ved siden af en (mulig) anden årsag for at ordinere dialyse I dag (valgfri).  Sæt et ”X” ved siden af evt. andre årsager som har påvirket din beslutning om at ordinere dialyse I dag (valgfri).  Tilføj gerne kommentarer.  Læg svaret i min kasse.  Der er ingen ”rigtige” eller ”forkerte” svar. Dine svar er fortrolige.  Selv om du ordinerer den først dialyse til start om fx én eller tre uger, udfyldes skemaet med dags dato. | | | | | |
| **Klinisk** |  | Social (hvad?) | | |  |
| Lungestase |  | Praktisk (hvad?)  (fx. kateterbeskyttelse) | | |  |
| Hypertension |  |  | | |  |
| Pericarditis |  |  | | |  |
| Ødem/Overhydrering |  | **Biokemisk** | | |  |
| Hjertesymptomer |  | Høj P-Creatinin | | |  |
| Træthed |  | Høj Karbamid | | |  |
| Anoreksi |  | Lav GFR | | |  |
| Kvalme/opkastning |  | Høj Kalium | | |  |
| Kakeksi/vægttab |  | Acidose | | |  |
| Kløe |  | Lav calcium | | |  |
| Insomnia |  | Høj Calcium | | |  |
| Depression |  | Høj Phosphat | | |  |
|  |  | Faldende GFR | | |  |
| Andet (hvad?) | | | | | |
| Kommentarer | | | | | |
